# Myxobacterial Predation: A Standardised Lawn Predation Assay Highlights Strains with Unusually Efficient Predatory Activity

**DOI:** 10.3390/microorganisms11020398

**Published:** 2023-02-04

**Authors:** Allison S. Zwarycz, David E. Whitworth

**Affiliations:** Department of Life Sciences, Aberystwyth University, Aberystwyth SY23 3DD, UK

**Keywords:** antimicrobial bacteria, lawn assay, microbial ecology, nutrient acquisition, phylogeny, phytopathogens

## Abstract

Myxobacteria prey upon a broad range of microorganisms. Lawn assays are commonly used to quantify myxobacterial predation—myxobacterial suspensions are spotted onto prey lawns, and monitored via spot expansion. The diversity in motility behaviours of myxobacterial strains and differing assay protocols in myxobacteriology laboratories led us to develop a highly-specified assay, which was applied to 28 myxobacterial strains preying on seven phytopathogenic prey species. Generally, prey organisms showed no qualitative differences in their susceptibility/resistance to myxobacterial predation. For most myxobacteria, prey did not stimulate, and in ~50% of cases actively hindered colony expansion. Only ~25% of predator/prey strain combinations exhibited greater colony expansion than in the absence of nutrients. The activity of predatory strains against different prey correlated, implying effective predators may have relatively non-specific predation mechanisms (e.g., broad specificity proteases/lipases), but no correlation was observed between predatory activity and phylogeny. Predation on dead (but intact) or lysed prey cells gave greater colony expansion than on live prey. Occasional strains grew substantially faster on dead compared to lysed cells, or vice-versa. Such differences in accessing nutrients from live, dead and lysed cells indicates there are strain-specific differences in the efficiencies/machineries of prey killing and nutrient acquisition, which has important implications for the ecology of myxobacterial predators and their prey.

## 1. Introduction

Myxobacteria (phylum *Myxococcota*) are microbial predators, and can grow and reproduce using nutrients obtained solely from live prey. The molecular details of their predatory mechanisms are relatively poorly understood; however, they are known to secrete a plethora of antimicrobial metabolites and proteins, both directly into the extracellular milieu and also packaged within ‘predatory’ outer membrane vesicles (OMVs) [1,2,3,4]. The accumulation of extracellular toxins around myxobacteria is thought to kill nearby prey cells, releasing nutrients into the public commons for myxobacteria to harvest, potentially making predation a cooperative process [5,6]. Recent evidence has also suggested that prey killing by myxobacteria can occur through contact-mediated transport of toxins into prey cells [7,8].

Myxobacteria predation assays are a common practice used to establish the predatory nature of myxobacteria strains against prey organisms. However, there is little consistency across predation assays used. Although not an exhaustive review of myxobacteria predation experiments, Supplemental Table S1 summarises a diverse set of experimental approaches from the literature, including mainly lawn, spot, streak and liquid assays, as well as a few less-common assays. Lawn assays, in general, spot a cell suspension of myxobacteria predator onto a lawn (high cell density) of prey. Spot assays tend to spot either a small amount of myxobacteria onto a spot of prey or vice versa. Streak assays create a rectangular patch of prey cells by streaking, and spot myxobacteria next to or near the streak. Liquid assays measure the surviving prey in a liquid medium predation experiment by plating onto selective media. Recently, single-cell assays have also been developed to view predation in real-time at the single cell level [9]. However, while single-cell predation assays visualise cell-cell transfer and lysis involving individual cells [7,10], group predation provides a community-level picture, and is typically assayed using spot plate or lawn predation assays (Supplemental Table S1).

The prey spot plate assay was originally designed to isolate myxobacteria from environmental samples [11]. Prey spot assays involve spotting a volume of prey cells onto an agar plate and allowing the spot to dry. Then, a small volume of predator cells is spotted onto the prey spot. Displacement/lysis halo diameters are measured after a given time interval [12,13,14]. In a lawn assay, prey cells are evenly spread onto the entire surface of an agar plate and allowed to dry. Then, plates are spotted with a small volume of predator cells and displacement/lysis halo diameters are measured. Prey in lawn assays are typically spread at a higher cell density than prey in the spot assay [15]. The main advantage of the lawn assay is that it is not restricted to the size of the prey spot, as is the case for the prey spot assay. In addition, myxobacteria are able to swarm on lawn assays, which allows for the calculation of prey-specific swarm rates [16]. Both methods have benefits and drawbacks, and ultimately the appropriateness of each assay depends on what research question is being asked. Some studies simply want to know if predation is occurring, while others are interested in comparing the predatory capacity of strains.

Even considering a single type of predation assay, there is considerable variation in how the assays are performed. Measured outputs of reported predation assays can vary considerably, as can important assay conditions. In some cases, the diameter of the swarm or lysis zone is measured, while in other cases a decrease in CFU (colony forming units) of prey is counted [17,18,19]. Other methods measure arbitrary lytic activity or patch encounter frequency [20,21,22]. In addition to measurement methods, the incubation conditions (temperature, duration) can be very different, as can the combinations of predator and prey used (Supplemental Table S1), which can have a huge influence on the outcome of predation assays [23,24]. Finally, few studies employ controls for motility, which is important for distinguishing the difference between movement and predation. Myxobacteria can move over surfaces by gliding motility, and swarms will migrate outwards in search of nutritional sources. The rate of this expansion depends on the type of medium used (nutrient vs. non-nutrient), the firmness of the surface (% agar), as well as the nature of the available prey.

Other limitations become apparent when considering the full diversity of predation assays. For example, relating myxobacterial predation to a positive control of the model myxobacterium *Myxococcus xanthus* DK1622, assumes that this organism is an optimal predator, which has been shown to not be the case in other studies [23,24,25]. In a similar respect, using *E. coli* as a control prey organism is also a peculiar choice, presumably born mostly out of convenience in the laboratory rather than any ecological or application relevance [26]. Assays using growth on nutrient medium as a control for motility do not account for inherent outward expansion of colonies in the absence of nutrients [16]. Finally, although convenient to assume that prey killing and lysis is taking place wherever myxobacteria expand over prey lawns, such an assumption can be misleading, as expansion may instead be indicative of other non-predatory phenomena (such as changes in motility).

Despite being widely used, there is no standard predation plate assay protocol for myxobacteria [14,23,25,27], and variations between assays hinder direct comparison of results between studies. Here, we provide a highly specified protocol for a standardised lawn predation assay for measuring the predatory performance of myxobacteria, which is reproducible and simple, but adaptable. The protocol improves upon currently used assays as it compensates for the inherent motility of myxobacterial strains, allowing changes in motility specific to the presence of prey to be determined. The protocol was validated on 28 myxobacteria and seven phytopathogenic prey organisms, and then adapted to characterise three aspects of the predatory process (killing, lysis of the prey cell, and nutrient consumption), by using live prey, intact dead prey cells, and lysed prey cells as nutrient sources in the assays.

## 2. Materials and Methods

### 2.1. Myxobacterial Strains

All myxobacteria (Table 1) were maintained at room temperature for up to four weeks on agar plates before re-plating and stored long-term as agar blocks at −80 °C. Myxobacteria were grown using three types of media, solidified if necessary with 15 g/L agar (Fisher): CY (3 g/L casitone (Gibco), 1 g/L yeast extract (Fisher), 1 g/L CaCl_2_.2H_2_O, pH 7.2), VY/2 (5 g/L Baker’s yeast, 0.5 g/L cyanocobalamin, 1 g/L CaCl_2_.2H_2_O, pH 7.2), and AMB (5 g/L soluble starch (Fisher), 2.5 g/L casitone, 0.5 g/L MgSO_4_.7H_2_O, 0.25 g/L K_2_HPO_4_). Plates were typically incubated between 20 and 30 °C, for a minimum of seven days and a maximum of three weeks, while liquid cultures were grown at 30 °C, 180 rpm for five to seven days, or until a specific OD_600_ was attained. TPM was used as a non-nutrient medium (10 mM Tris pH 7.6, 1 mM KPO_4_, 8 mM MgSO_4_).

### 2.2. Prey Strains

Because of the potential for using myxobacteria as biological control agents, seven phytopathogenic prey organisms were chosen, representing a taxonomically diverse set of organisms that infect a variety of economically important plant hosts (Table 2). Gram-negative organisms were grown in LB broth (10 g/L tryptone (Fisher), 10 g/L NaCl, 5 g/L yeast extract) for 18–24 h at 37 °C, 180 rpm. Gram-positive and fungal organisms were grown in Tryptone Soy Yeast Extract (17 g/L tryptone, 3 g/L soya peptone (Oxoid), 6 g/L yeast extract, 5 g/L NaCl, 2.5 g/L K_2_HPO_4_, 2.5 g/L glucose) or YEPS (10 g/L yeast extract, 20 g/L tryptone, 10 g/L saccharose (Fisher)), respectively, for 40 h at 30 °C, 180 rpm. Strains were maintained at 4 °C for up to two weeks on agar plates before re-plating and stored long-term as liquid glycerol stocks at −80 °C.

### 2.3. Standardised Lawn Predation Assays

#### 2.3.1. Preparation of Myxobacteria and Prey Cells

Myxobacteria were grown to exponential phase (OD_600_ ≈ 1) in AMB medium. Myxobacteria cells were then sedimented by centrifugation (4000× *g* for 30 min), washed three times in TPM, centrifuged again and resuspended in a suitable volume of TPM to give a calculated OD_600_ value of 2.5. This cell density ensured that on the final day of the assay, the swarm diameter (displacement) could be measured accurately even for slow growing/moving myxobacteria, while displacement did not exceed the dimensions of the petri dish for faster growing/moving myxobacteria.

Prey cells were cultured in their optimal nutrient medium until growing exponentially (OD_600_ ≈ 1) then washed and resuspended in TPM as above for myxobacteria, except to a higher calculated OD_600_ value of 6.5. This cell density was required to generate a visible lawn that covered an entire petri dish. OD_600_ was used to specify the density of prey cell preparations rather than CFUs or virtual colony counts (VCCs), because cell size can vary between prey, which is not accounted for by measuring CFUs or VCCs. Instead, optical density measures the physical space occupied by cells. In addition, OD values are much faster to measure than CFUs or VCCs. An immediate measure of cell density/number is needed before starting the assays so that cultures can be normalised to the required density and the assay started without delay. To ensure that normalising by OD_600_ for diverse prey species did not generate assays with substantially different numbers of cells and therefore predator:prey ratios, the cful/mL/OD_600_ values were assessed and found to only vary by a maximum factor of 1.29 between different prey species.

#### 2.3.2. Performing the Assays

To create a lawn, 500 μL of prey cell suspension was spread evenly on the surface of TPM agar (e.g., 1.5% agar in TPM) petri dishes and allowed to fully dry at room temperature. To initiate the assay, 2 μL of the concentrated myxobacteria cell suspension was spotted onto the prey lawn in quadruplicate—one spot in the centre of each quadrant of the petri dish (resulting in an approximate ratio of 125 myxobacteria to 1 prey over the area of the myxobacteria spot). In addition, the same myxobacteria cell suspensions were spotted in quadruplicate onto two control plates: TPM agar and AMB agar (1 spot per plate), both without prey lawns. The predator spots were allowed to dry and then incubated at 30 °C. (To avoid potential competition between different myxobacterial genotypes, only one strain of myxobacteria was used on each plate.)

#### 2.3.3. Analysis of Assay Measurements

Mean displacement measurements were used to calculate two further variables; ‘displacement ratio’ and ‘prey-specific swarm rate’. The displacement ratio Equation (1), corrects the displacement measurements observed for myxobacterial spots on prey lawns (on TPM) to take into account intrinsic myxobacterial colony expansion, by subtracting the displacement observed on TPM plates lacking a prey lawn. As the rate of intrinsic spot expansion is stimulated by the presence of nutrients, the ‘corrected displacement’ on prey lawns is then normalised against corrected displacement on AMB nutrient plates.

(1) $$\mathrm{Displacement}\;\mathrm{Ratio}=\frac{{\mathrm{Displacement}}_{\mathrm{prey}}-{\mathrm{Displacement}}_{\mathrm{TPM}}}{{\mathrm{Displacement}}_{\mathrm{AMB}}-{\mathrm{Displacement}}_{\mathrm{TPM}}}$$

where Displacement_prey_ is the mean displacement (mm) on prey lawn, Displacement_TPM_ is the mean displacement on TPM medium and Displacement_AMB_ is the mean displacement on AMB medium. The ‘prey-specific swarm rate’ Equation (2), divides the mean corrected displacement by the number of elapsed days.

(2) $$\mathrm{Prey}\;\mathrm{Specific}\;\mathrm{Swarm}\;\mathrm{Rate}\;=\frac{{\mathrm{Displacement}}_{\mathrm{prey}}-{\mathrm{Displacement}}_{\mathrm{TPM}}}{\mathrm{Days}\;\mathrm{elapsed}}$$

All data analysis and statistical tests were performed in SPSS (v. 27). Student’s *t*-tests and ANOVA were used to measure significant differences between displacement on given prey and varying prey conditions. Tukey’s post-hoc tests were used to identify group significant differences.

### 2.4. Elaborations of the Standard Assay

As alternatives to live prey, dead (but intact) and lysed prey were also used for predation plate assays. For both dead and lysed conditions, washed cells were incubated in 70% ethanol for 30 min on ice, followed by three TPM washes. To generate lysed cells, suspensions were then subjected to sonication for two minutes at 30 W. To confirm killing and lysis of cells, dead and lysed cells were plated on appropriate nutrient medium, incubated overnight and observed under 40X magnification. Killed and lysed cells were plated to generate lawns for the predation assay in the same manner as live prey. Dead (but intact) lawns were very similar in character to those of live prey, but lawns of lysed cells were more transparent.

### 2.5. Pan-Genomics, ANI Calculations and Clustering Dendrograms

The identification of orthologous gene families in the myxobacterial pan-genome was achieved using Pirate v. 1.0.3. [34]. The predicted subcellular locations of gene products were determined using PSORTb [35], potential secretion mechanisms were assessed using SignalP [36], and COG and KEGG groupings were established using EggNOG [37]. To determine the average nucleotide identity (ANI) an ANI-based all-vs-all matrix was constructed using the ANI-Matrix genome calculator [38]. Hierarchical clustering using the complete linkage method, generation of dendrograms and tanglegrams, and correlation coefficient calculations were performed in R using the dendextend library [39].

## 3. Results

### 3.1. Development of a Standardised Lawn Plate Assay for Myxobacterial Predation

A standardised lawn predation assay for myxobacteria is provided in the Section 2. The protocol was developed to work well for a large range of predator and prey strain combinations despite the variability in strain characteristics. For instance, myxobacterial species have differing optimal growth temperatures, typically between 20 and 32 °C, and most myxobacteria do not grow well at higher or lower temperatures [28,29]. A temperature of 30 °C was ultimately chosen as it gave near-optimal growth for the majority of myxobacteria tested. Similarly, the motility of myxobacterial strains can vary considerably between strains as it depends on the interplay of multiple motility engines, which themselves depend on the proximity of other myxobacteria and prey cells. To correct for the strain-specific inherent outward movement of myxobacterial colonies, control plates with myxobacteria spotted onto nutrient (AMB) and ‘non-nutrient’ (TPM) agar are used in the assay. (Although agar itself usually contains trace amounts of nutrients, they are not concentrated enough to support growth of myxobacteria or prey, and TPM plates are hence referred to as non-nutrient here). Expansion on nutrient agar reflects near-optimal growth and displacement, while expansion on non-nutrient agar captures inherent motility behaviour.

### 3.2. Assaying Predation of a Diverse Selection of Myxobacteria and Prey

**Figure 1:**
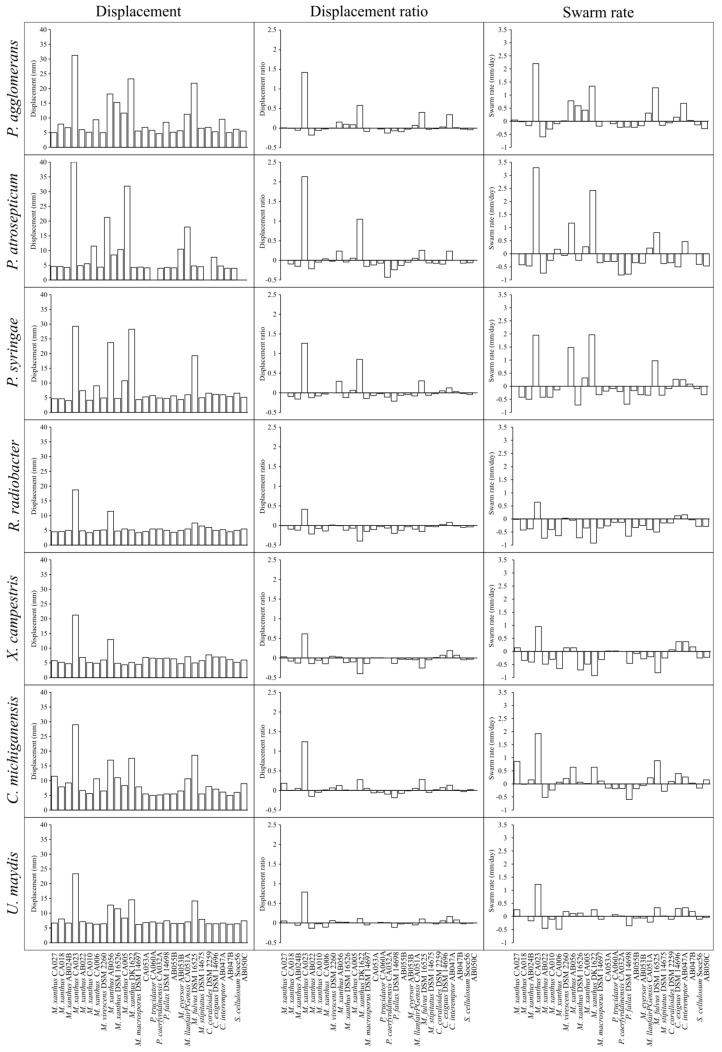
Myxobacteria displacement, displacement ratio and swarm rate on seven phytopathogenic prey organisms. Displacement (**left**) is the diameter (mm) of the advancing swarm edge on day eight on a lawn of prey. Displacement ratio (**middle**) is calculated using Equation (1) and compensates for differences in strain-specific intrinsic swarm expansion with/without nutrients. Swarm rate (**right**) is measured as the rate (mm/day) of displacement on a lawn of prey minus the rate of displacement on non-nutrient TPM agar. Myxobacteria are ordered according to ANI similarity. From left to right, strains are: CA027, CA018, AB024B, CA023, AB022, CA010, CA006, DSM 2260, AB056, DSM 16526, CA005, DK1622, DSM 14697, CA053A, CA060A, CA032A, DSM 14698, AB055B, AB053B, CA051A, DSM 16525, DSM 14675, DSM 2259, DSM 14696, AB047A, AB047B, So ce56, AB050C.

To test the performance of the assay, lawn assays were performed for 28 myxobacterial strains on seven phytopathogenic prey organisms (Table 1 and Table 2, respectively), characterising displacement (spot diameter), displacement ratio (as defined in Equation (1)) and prey-specific swarm rate (Figure 1). As with medically relevant pathogens [23], all myxobacterial strains were able to prey upon every phytopathogen tested, exhibiting clearance of the prey lawn where spotted. After incubation, some myxobacteria exhibited noticeably larger displacement values than the average myxobacterium, but this varied with prey species. For instance, after eight days’ incubation, *M. xanthus* CA023 and *M. xanthus* AB056 spots had grown to a diameter two to five-times greater than most other strains. *M. xanthus* DK1622 also exhibited unusually large displacement values, but only for three of the seven prey (*Pantoea agglomerans*, *Pectobacterium atrosepticum* and *Pseudomonas syringae*).

Displacement ratios were calculated, and for the majority of predator-prey combinations the displacement ratio was close to zero, albeit with some notable exceptions. Some predator-prey combinations with large spot displacements did give high displacement ratios (e.g., *M. xanthus* CA023), however others did not (e.g., *M. xanthus* AB056), suggesting that differences in intrinsic motility could underlie some of the larger spot displacements, rather than superior predation *per se*. This suggestion was supported by swarm rate calculations, which showed a great deal of variability between myxobacterial strains. It was noticeable that of the 194 predator-prey combinations tested, only five combinations had a displacement ratio >1 (*M. xanthus* CA032 with *P. agglomerans*, *P. atrosepticum*, *P. syringae* and *Clavibacter michiganensis* and *M. xanthus* DK1622 with *P. atrospeticum*). A displacement ratio >1 indicates that displacement on prey exceeds even that observed on nutrient plates. In comparison, seven myxobacterial strains (*Myxococcus eversor* AB053B, *Myxococcus* sp. AB055B, *M. xanthus* strains CA018, AB022, CA010, *Pyxidicoccus fallax* DSM14698 and *Pyxidicoccus* sp. CA053A) exhibited displacement ratios that were less than or equal to zero on all seven prey tested, indicating particularly poor predatory activity.

A large proportion of predator-prey combinations gave a negative prey-specific swarm rate indicating that the presence of prey cells had impeded the intrinsic outward swarming of the myxobacteria (Figure 1). Comparing the displacement of myxobacterial spots on AMB nutrient plates with displacement on TPM non-nutrient plates (Supplemental Figure S1), shows that in the absence of prey, nutrients increase the rate of outward swarming for every myxobacterial strain tested (between two-fold and ten-fold, depending on the strain). As successful predation liberates nutrients, we would expect myxobacteria to exhibit greater displacement on prey lawns than in the absence of nutrients, however this seems to actually be observed for only a small minority of predator-prey combinations which have positive prey-specific swarm rates (Figure 1).

### 3.3. Categorisation of Myxobacterial Prey-Specific Predatory Behaviours

**Figure 2:**
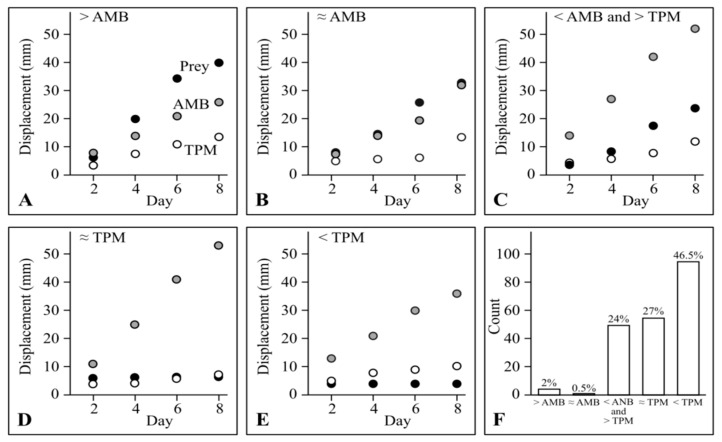
Displacement patterns of myxobacteria. (**A**) CA023 on *P. atrosepticum*, (**B**) *M. xanthus* DK1622 on *P. atrosepticum*, (**C**) *M. xanthus* AB056 on *P. syringae*, (**D**) *S. cellulosum* Soce56 on *U. maydis*, (**E**) *P. fallax* DSM 14698 on *P. atrosepticum*. In (**A**–**E**), black, grey and white circles indicate displacement on prey, AMB medium and non-nutrient TPM medium, respectively. (**F**) The count (out of 194) and percentage of predator/prey categorization.

As no myxobacteria exhibited displacement on TPM that exceeded displacement on AMB, the predatory behaviour of myxobacterial strains in the lawn assay can be objectively categorised by comparing their displacement on specific prey with their displacement on TPM and AMB after eight days. Displacements ‘exceeding AMB’ and ‘below TPM’ were defined as having mean values more than 1 mm above the displacement on AMB and more than 1 mm below the displacement on TPM, respectively. Displacement ‘between AMB and TPM’ was defined as displacement more than 1 mm above that observed on TPM and more than 1 mm below that observed on TPM. Finally, displacements ‘similar to AMB’ or ‘similar to TPM’ were defined as displacement within 1 mm of displacement on AMB or TPM, respectively. Examples of the five displacement categories are shown in Figure 2: exceeding AMB (Figure 2A), similar to AMB (Figure 2B), between AMB and TPM (Figure 2C), similar to TPM (Figure 2D), and below TPM (Figure 2E).

Almost half (46.5%) of all predator/prey combinations had displacement ‘below TPM’ (corresponding to displacement ratios < 0, with negative prey-specific swarming rates). Around a quarter (27%) displaced ‘similar to TPM’, and another 24% displaced ‘between AMB and TPM’. Only 0.5% and 2% displaced ‘similar to AMB’ or ‘exceeding AMB’, respectively (Figure 2F), highlighting those strains which are particularly effective at liberating nutrients from prey lawns (displacement ‘exceeding AMB’ corresponds to a displacement ratio > 1).

### 3.4. Correlations of Predatory Performance

**Figure 3:**
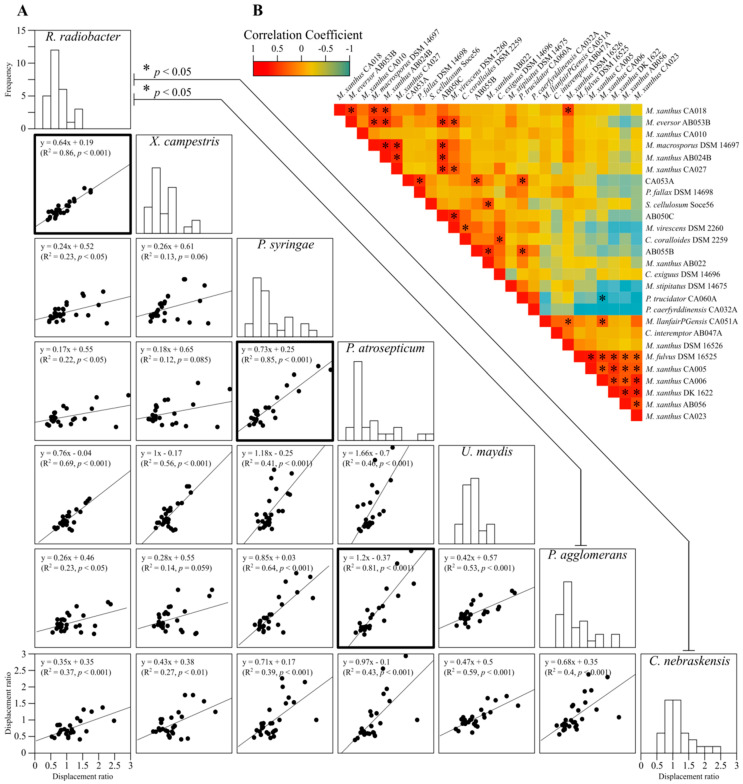
Pairwise comparisons of myxobacteria predation. (**A**) linear regressions and correlations between displacement ratios for pairs of prey organisms, and histograms of displacement ratios for each prey. Lines between histograms have a significantly different mean (ANOVA F(6, 180) = 2.624, *p* = 0.018). Bold boxed scatter plots indicate a significant linear regression (R^2^ > 0.75 and *p* < 0.001). (**B**) correlations in displacement ratios between myxobacteria strains, where warm and cool colours indicate high and low correlation coefficients, respectively. Boxes with asterisks indicate significant correlations (*p* < 0.05), all of which were positive except for CA006/CA060A.

To identify potential relationships between predatory strains and between prey strains (i.e., predator vs. predator and prey vs. prey), pair-wise comparisons of displacement ratios were performed, including linear regressions and correlations (Figure 3). For comparisons between prey susceptibility to myxobacterial predation, all but three of the 21 comparisons were significantly similar, however, only three possessed coefficients of variation (R^2^) above 0.75 (Figure 3A, bold boxes). These results confirm that considered as a whole, myxobacteria prey similarly on a wide variety of phytopathogenic organisms, regardless of the prey’s phylogeny.

Incubation conditions were kept constant throughout the duration of the assay and minimising the amount of time taken to record displacement. Measurements of displacement (spot diameters, in mm) were recorded at regular intervals for each spot. Typically, myxobacteria grow uniformly outward with time, nevertheless, two cross-sectional (orthogonal) measurements were taken for each spot and averaged, to compensate for potential anisotropy. Measurements were taken every two days for eight days.

Some myxobacteria share similar predatory profiles of activity against prey, giving a significant positive correlation coefficient (shown red in Figure 3B). On a few occasions, predators share a negative correlation (e.g., CA006 and CA060A), which suggests that where one predator has a high displacement ratio on one or more prey, the opposite is true for the other predator (shown in blue in Figure 3B). There is just a single case where a negative correlation was significant (*p* < 0.05), for *Pyxidicoccus trucidator* CA060A and *M. xanthus* CA006. The predators generally display a predatory profile that does not correlate significantly with that of other predators (shown in yellow/orange in Figure 3B). While prey are similarly killed by a set of myxobacteria, it appears that each predator has a selective ability to prey on specific phytopathogens to varying degrees.

**Figure 4:**
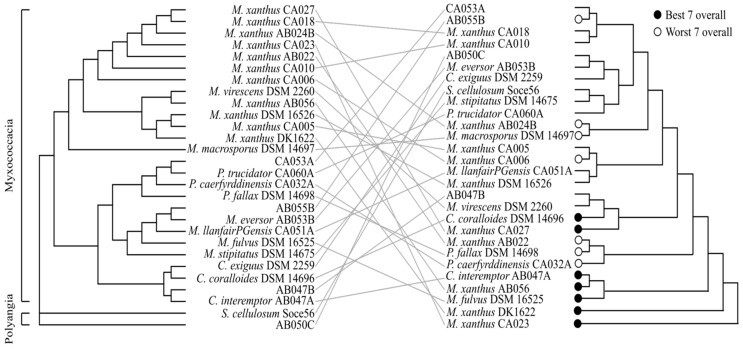
ANI distance tree and predation performance tanglegram. ANI similarity (**Left**) does not reflect predation performance (**Right**). ANI distancing clustered myxobacteria into two classes, *Myxococcacia* and *Polyangia*, and separated the *Myxococcacia* by genera. Hierarchical clustering of predation performance: black and white points indicate the ‘best’ and ‘worst’ seven overall predators, respectively.

To investigate any relationship between phylogeny and predatory behaviour, a distance tree was generated using genome-wide ANI (average nucleotide identity) values, which recapitulates the known taxonomy of myxobacteria (Figure 4, left). A dendrogram representing predatory behaviour was created in R using the “complete” agglomeration method, inputting displacement ratios for predation against all seven prey (Figure 4, right). In the predatory behaviour dendrogram, although some members of the same genus appear to cluster near one another, overall phylogeny does not agree well with predatory behaviour, as indicated by the tanglegram (Figure 4). In addition, the lack of discrete clustering of the best and worst predators (according to the mean displacement ratio across all prey) is indicative of the diversity in predatory ability against different prey. In most cases displacement of individual myxobacterial strains is high on one or two prey but average or low on the others.

Predatory ability may be influenced by the presence or absence of common gene families in the best predators. To assess this, a pan-genome analysis was performed, which identified a small number (593) of core gene families present in all 28 organisms. No gene families were found to only be present in the best seven predators. Indeed, no gene families present in two or more predator genomes were found to only be present in any number of the top predators when ranked by mean displacement ratio. Similarly, there are no gene families present in the worst 21 predators that are not also present in at least one of the top seven predators. These observations suggest that predation performance is not dictated by phylogeny, either as assessed by ANI or by the presence/absence of common gene families, supporting the conclusions of previous studies with different prey [23]. In addition, our analysis shows that the presence or absence of gene families cannot explain observed patterns of predatory ability.

### 3.5. Increased Displacement Is Observed on Dead and Lysed Cells Compared with Living Prey Cells

**Figure 5:**
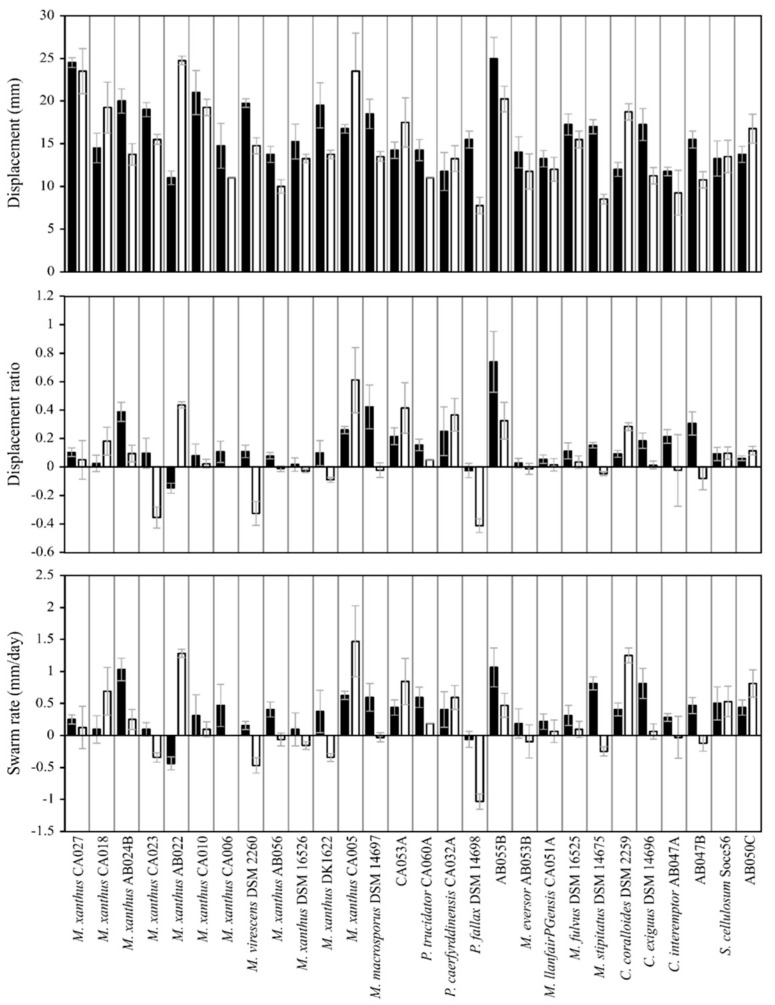
Displacement (**top**), displacement ratio (**middle**) and swarm rate (**bottom**) of myxobacteria strains on *Pectobacterium atrosepticum*. Black and white bars reflect dead and lysed prey cells, respectively. Error bars represent standard deviation, where *n* = 4 for each myxobacterium.

**Figure 6:**
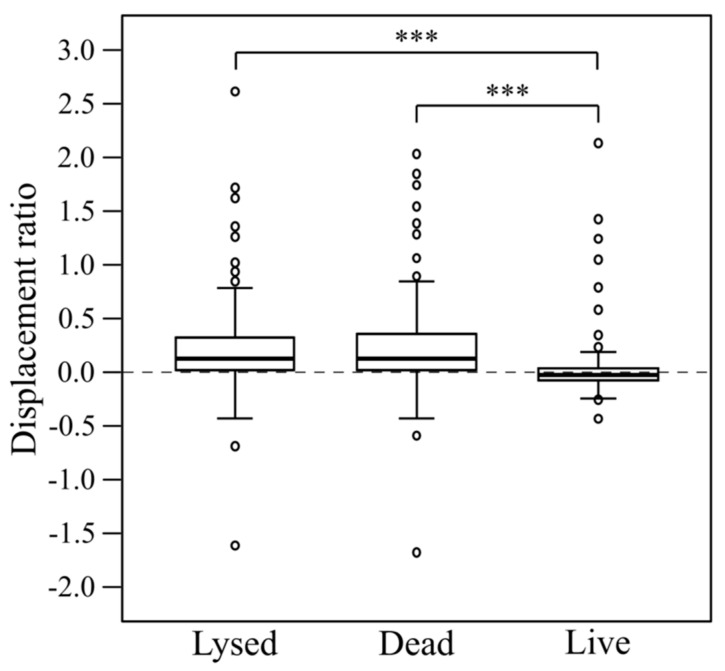
Comparison of myxobacterial displacement ratios obtained on lysed, dead and live prey cells. Asterisks (***) signify highly significant differences (*p* < 0.001, *n* = 28).

To investigate whether the physiological status of the prey affected the activity of myxobacterial predators, and to illustrate a simple adaptation of the standardised lawn assay protocol, the assay was used to assess predation on ethanol-killed ‘dead’ and sonicated ‘lysed’ prey cells, for the same 28 myxobacteria and seven prey strains used previously. The results for *P. atrosepticum* are shown as an example in Figure 5 and those for the other six prey as Supplemental Figures S2–S7. When considering all myxobacteria on all prey as a single population, there is a statistically significant difference between predator displacement ratios on dead (M = 0.03, SD = 0.3), lysed (M = 0.24, SD = 0.4) and live (M = 0.21, SD = 0.4) prey cells, as determined by one-way ANOVA (F(2, 585) = 17.398, *p* < 0.001). A Tukey HSD (honestly significant difference) post-hoc test confirmed the significant difference is between dead/live prey and lysed/live prey (both with *p* < 0.001), but not between dead/lysed prey cells (Figure 6). This suggests that in general, living prey actively hinder predation by myxobacteria and that myxobacteria are equally well able to feed on dead and lysed prey cells.

Nevertheless, for some individual prey organisms (*P. agglomerans*, *P. atrosepticum*, *P. syringae* pv. *tomato* and *Ustilago maydis*), myxobacterial predatory activity is significantly affected by whether those prey are provided as dead or lysed cells (Table 3). Differences in myxobacterial predation metrics on dead/lysed cells are also observed when comparing between different prey species. Whether provided as dead or lysed cells, *U. maydis* gave significantly higher values for all three predation metrics than any other prey strains (Table 4). Conversely, dead *C. michiganensis* subsp. *nebraskensis* cells gave significantly lower myxobacterial displacement values than on any other prey. Additional pairwise differences in predatory behaviour on different prey species are also observed, but with slightly more observed for lysed prey than dead prey (Table 4).

Some myxobacterial strains also exhibited significantly different predation behaviours when comparing between lysed and dead prey, in a prey-specific manner (Figure 5 and Figure S2–S7). For instance, eight, 17, 13, 14, 12, 13 and 19 myxobacterial strains exhibited significantly different behaviour on dead vs. lysed cells for all three metrics, with *P. agglomerans*, *P. atrosepticum*, *P. syringae*, *Rhizobium radiobacter*, *Xanthomonas campestris*, *C. michiganensis* and *U. maydis* prey, respectively. No myxobacterial strain exhibited statistically significant differences between dead and lysed prey cells for all seven prey strains tested (for any of the three predation metrics). However, five strains (*M. xanthus* AB022, *M. xanthus* AB056, *Myxococcus virescens* DSM 2260, *M. virescens* AB055B and *Corallococcus coralloides* DSM 2259) exhibited a significant difference between their responses to live and dead prey for six out of seven prey species tested, in at least two of the three metrics. Where significant differences between dead/lysed were observed, there was no pattern in whether the predation metric was greater on dead or lysed cells (Figure 5 and Figure S2–S7).

Taken together, these observations indicate that while there is no general difference in myxobacterial predation on dead and lysed prey cells, there are some prey and predator strains (and specific combinations thereof), for which predatory activity is highly dependent on whether the prey cell has been lysed or not. In some cases, predation is greater when prey cells are lysed, while in other cases predation is greater when prey cells are intact.

## 4. Discussion

Over the years there has been considerable variation in how different research laboratories have deployed myxobacterial predation lawn assays (Supplemental Table S1). In addition, researchers have used a wide range of predatory myxobacterial strains and a greater diversity of prey strains, which behave very differently from one to another on agar plates. To improve consistency and comparisons between assays, we developed a highly specified assay which normalises outward expansion (displacement) of a myxobacterial spot on a lawn of prey, against expansion on agar in the presence and absence of nutrients. Myxobacterial colonies expand outwards at an inherent rate due to the presence of active gliding motility systems. Growth of a population can also lead to the expansion of a colony, so by normalising against displacement in the absence of nutrients (governed by inherent motility) and in the presence of nutrients (governed by inherent motility plus population growth), the calculation of the displacement ratio enables benchmarking of each strain’s response to prey against its predation-independent growth and motility. Further improvements in the assay methodology could be envisaged. For instance, our approach is highly dependent on accurate measurements of displacement, which is currently measured at the leading edge of the swarm in millimetres. However, it can be extremely difficult to robustly determine the location of the leading edge, as single predator cells infiltrate into prey colonies ahead of the leading edge [40,41].

As can be seen in Figure 1, the profile of displacement ratios appear similar for every prey organism used, despite including fungi, Gram-negative and Gram-positive bacteria in our assays. In every case, the majority of myxobacterial strains had displacement ratios close to zero, but with occasional strains doing particularly well (or badly) on specific prey. It has been previously established that myxobacteria predate differently depending on the Gram-stain of the encountered prey, with Gram-positive organisms tending to resist predation more than Gram-negative bacteria [14,27]. However, when considering all 28 myxobacteria strains, we do not observe any significant difference in predation susceptibility between prey organisms in our data, nor any difference between predation on bacterial prey or the fungus *U. maydis*. Mean displacement ratios for each prey were only significantly different when comparing Gram-negative *R. radiobacter* with *P. agglomerans* (another Gram-negative bacterium) and *Clavibacter mighiganensis* (Gram-positive). All other comparisons were not significantly different.

It is somewhat surprising that the displacement ratios of most predator-prey combinations are close to zero. The availability of nutrients in the form of prey, would be expected to stimulate colony expansion of all predator and prey combinations relative to that seen on non-nutrient agar, by fuelling population growth. However, that only seems to be the case for a minority of predator-prey combinations. In many cases, the lawn of prey actually reduces the rate of outward spot expansion, as shown by calculating prey-specific swarm rates (Figure 1), suggesting that in general, live prey resist the outward expansion of myxobacterial colonies. Nevertheless, around a quarter of predator-prey combinations had positive displacement ratios, indicating that colony expansion was greater in the presence of prey than on non-nutrient agar, and providing evidence of active predation.

While there was little difference between prey in terms of their susceptibility to myxobacterial predation, there was considerable variation between the activity of myxobacterial strains against particular prey, with the profile of predatory activity even being negatively correlated between some strains (Figure 3). The pattern of similarity in myxobacterial predatory activity did not correlate with phylogeny (whether assessed via the presence/absence of gene families, or through genome-wide ANI comparisons). Five combinations of predator and prey strains gave displacement ratios greater than 1, suggesting particularly efficient predation by those predators on those prey. As taxonomically distant prey organisms exhibit very little difference in their susceptibility/resistance to myxobacterial predation, it seems likely that particularly effective predation is observed when predatory strains uniquely have digestive enzymes able to act on major components of prey biomass, and/or unique secondary metabolites with high specificity towards particular prey. The myxobacterial pan-genome is large and open, with individual strains having highly individualised accessory genomes [29,42], which makes such scenarios quite plausible.

Previously, a genome-wide association study was used to identify candidate myxobacterial genes whose presence/absence correlated with good/bad predation on human pathogenic prey organisms [43]. Although here we could find no clear link between the presence/absence of gene families and predatory performance against phytopathogenic prey, we also compared our predation data with proteomic datasets [31] that were available for the OMVs of ten of the *M. xanthus* strains used in this study. Of the ten strains for which OMV proteome data were available, four predators (*M. xanthus* AB056, CA023, CA027, and DK1622) were the best (as judged by mean displacement ratios), whether considering fungal, Gram-negative and/or Gram positive bacteria. Only one OMV protein was found in the OMVs of those four strains and not the other six—PrtB, protease B (encoded by MXAN_2791). Another eight proteins were found in three of the four best predators and no others, of which seven had only vague annotation. However, the eighth was encoded by MXAN_2814, which is annotated as an N-acetylmuramoyl-L-alanine amidase, which is a peptidoglycan degrading enzyme. Intriguingly, MXAN_2814 is found in an operon upstream of the gene for GAPDH, which has been implicated in enhancing the predatory activity of OMVs [1,44]. Proteases and peptidoglycan hydrolases have been shown to contribute to OMV-mediated predation previously [45] making them attractive candidates for further study in myxobacterial predation.

Predation can be thought of as a triphasic process: prey killing, cell lysis for nutrient liberation and nutrient acquisition/use [41]. At the single-cell level it appears that the first wave of myxobacteria may be responsible for killing and lysing cells before moving on to other cells, in a phenomenon termed lysis-leave. In addition, myxobacteria appear to ‘pause’ when first contacting prey cells (*E. coli*) which has been suggested to allow the myxobacterium time to synthesize and release lytic metabolites [9]. It would be predicted that a ‘pause’ on encountering prey during the first phase of predation would lead a predatory colony to exhibit a lower displacement with live prey (and possibly dead prey) than with lysed prey cells. Similarly, a reduction in displacement might also be expected when comparing between displacement on dead and lysed cells due to the time required to break open the dead cell to access the nutrients within. A reduction in displacement was observed when comparing live prey with lysed prey, but not when comparing dead cells with lysed prey (Figure 6), suggesting that prey killing limits the rate of predation more than prey lysis or nutrient uptake.

The relative resistance of colony expansion by live prey could be due to prey cells acting as a physical barrier to myxobacterial movement, and/or because they possess active anti-predation mechanisms. However, the latter scenario seems more likely, as dead but intact prey cells do not appear to hinder myxobacterial displacement any moreso than lysed cells. If so, presumably the myxobacterial strains with high displacement ratios are able to evade the prey’s defences to an unusual extent.

Some individual myxobacterial strains exhibited clear preferences for dead or lysed prey, which differed depending on the prey organism. Such preferences were independent of phylogeny (of prey and predator) and there was no consistent pattern of preference for either dead or lysed prey cells. Among the prey, only *P. agglomerans* and *P. atrosepticum* belong to the same order, and even those two organisms shared little in common when comparing their dead/lysed predatory profiles (Figure 5 and Figure S2). Across all myxobacterial strains, *U. maydis* displayed consistently high displacement ratios compared to other prey, whether provided as dead or lysed cells (Supplemental Figure S7), however a pattern of only occasional preferences for dead/lysed prey cells was still seen.

Although the conditions used in our assays are unlikely to accurately emulate typical conditions in soil, the observed preferences for dead/lysed prey cells may have implications for microbial ecology and evolution. If some strains are better than others at acquiring nutrients from lysed cells, but are worse at killing and lysing cells, then in mixed populations they might appear as ‘cheaters’, exploiting strains that are more proficient at killing, but less efficient at nutrient uptake. While cheating has been observed at multiple points in the myxobacterial life-cycle, it has not yet been documented to happen during predation [46,47].

For six of the seven prey organisms, mean displacement ratios were higher when provided as dead cells rather than lysed cells. Only *P. syringae* gave increased mean displacement ratios when lysed rather than when provided as dead (but intact) cells, suggesting there is no particular cost associated with lysing dead cells. Increased myxobacterial displacement on dead cells rather than lysed cells could indicate that dead cells keep nutrients concentrated in a defined location, feeding those cells at the edge of the colony actively engaged in killing prey and fuelling colony expansion, while lysed cells release their nutrients to the environment allowing them to be transported away from where they were killed and reducing the benefit to cells at the vanguard of the advancing colony. Prey cell lysates may also directly interfere with myxobacterial motility, for instance by changing the viscosity of the surface. Presumably idiosyncrasies between myxobacterial strains in their ability to grow on different lysed prey might also reflect differences in their ability to digest or take up the cellular components of particular prey. Surprisingly, very few combinations of predator and prey gave displacement ratios above 1 when grown on lysed prey, indicating poorer growth than on AMB nutrient plates. Maybe the AMB had a more optimal balance of nutrients for myxobacterial growth than lysed prey cell material. It is also possible that not enough lysed cell material was added to provide the same amount of nutrient as available in AMB.

While differences in predator displacement on dead and lysed prey cells could be informative regarding the mechanisms of predation, it is difficult to extrapolate findings to myxobacterial predation in the wild. It is not yet clear how different strains of predators and/or prey are distributed between micro-niches within the soil. The relationship between prey cell lysis and killing is also not clear cut. Prey cell killing can occur before lysis in vitro, for instance when adding purified myxobacterial OMVs to prey cells [1], or when treating cells with ethanol, but if the natural mechanism of prey killing is via cellular lysis, then killing will also liberate nutrients, making it impossible to disentangle prey killing from lysis and nutrient uptake. It would also mean that some myxobacteria might never encounter dead but intact cells, making it difficult to interpret the relevance of our experimental results to predation in the wild. Previous studies have suggested that some predatory mechanisms target intracellular machinery, while others cause prey lysis by degrading prey peptidoglycan [24], implying that sometimes prey are killed without undergoing lysis, whilst at other times they die as a result of lysis.

In summary, the development of a standardised lawn predation assay has allowed us to characterise general features of myxobacterial predation, as well as highlight particularly good predators, rather than those which just appear good due to high intrinsic motility rates. Idiosyncrasies in preferences for live, dead and lysed prey have also been identified and deserve further investigation, as they could potentially bring novel antimicrobial enzymes and metabolites to light, increasing our mechanistic understanding of myxobacterial predation and the potential for exploitation as novel anti-infectives [48]. Despite the difficulties in extrapolating assay results to real-world situations, our experimental observations also suggest that in the wild, most combinations of strains would likely result in a slow but inexorable struggle between predator and prey. However, in a few predator/prey combinations, unique myxobacterial enzymes/metabolites can render prey defences ineffective, allowing unusually efficient and fast predation.

## Figures and Tables

**Table 1:** Myxobacteria strains used. DSMZ refers to strains obtained from the German Collection of Microorganisms and Cell Cultures. *Myxococcus llanfairPGensis is an abbreviation of Myxococcus llanfairpwllgwyngyllgogerychwyrndrobwllllantysiliogogogochensis*.

Class	Genus	Species/Strain	Reference/Source	Genbank Accession
Myxococcia	*Corallococcus*	*Corallococcus coralloides* DSM 2259	DSMZ	GCA_000255295
		*Corallococcus exiguus* DSM 14696	DSMZ	JAAAPK000000000
		*Corallococcus interemptor* AB047A	[28]	RAWM00000000
		*Corallococcus* sp. AB047B	[23]	-
	*Myxococcus/Pyxidicoccus*	*Myxococcus eversor* AB053B	[29]	JAAIXY01000000000
		*Myxococcus fulvus* DSM 16525	DSMZ	FOIB00000000
		*Myxococcus llanfairPGensis* CA051A	[30]	GCA_013336705
		*Myxococcus macrosporus* DSM 14697	DSMZ	GCA_002305895
		*Myxococcus stipitatus* DSM 14675	DSMZ	GCA_000331735
		*Myxococcus virescens* DSM 2260	DSMZ	FNAJ00000000
		*Myxococcus xanthus* AB022	[31]	VHLD00000000
		*Myxococcus xanthus* AB024B	[31]	SRLY00000000
		*Myxococcus xanthus* AB056	[31]	VHLB00000000
		*Myxococcus xanthus* CA005	[31]	SRLV00000000
		*Myxococcus xanthus* CA006	[31]	SRLU00000000
		*Myxococcus xanthus* CA010	[31]	VHLA00000000
		*Myxococcus xanthus* CA018	[31]	JAAEAG000000000
		*Myxococcus xanthus* CA023	[31]	JAAEAH000000000
		*Myxococcus xanthus* CA027	[31]	WBSK00000000
		*Myxococcus xanthus* DK 1622	[32]	GCA_000012685
		*Myxococcus xanthus* DSM 16526	DSMZ	GCA_900106535
		*Myxococcus* sp. AB055B	[23]	-
		*Pyxidicoccus caerfyrddinensis* CA032A	[29]	JAAIYA000000000
		*Pyxidicoccus fallax* DSM 14698	DSMZ	JABBJJ000000000
		*Pyxidicoccus trucidator* CA060A	[29]	JAAIXZ000000000
		*Pyxidicoccus* sp. CA053A	[23]	-
Polyangia	*Sorangium*	*Sorangium cellulosum* So ce56	[33]	AM746676
		*Sorangium* sp. AB050C	[23]	-

**Table 2:** Prey phytopathogen strains used.

Organism	Plant Host(s)	Order	Strain	Gram Stain
*Pantoea agglomerans*	*Pyrus communis*	Enterobacterales	NCPPB 1269	Negative
*Pectobacterium atrosepticum*	*Solanum tuberosum*	Enterobacterales	NCPPB 138	Negative
*Pseudomonas syringae* pv. *tomato*	*Solanum lycopersicum*	Pseudomonadales	DC 3000	Negative
*Rhizobium radiobacter*	*Cucumis sativus*	Rhizobiales	NCPPB 2655	Negative
*Xanthomonas campestris* pv. *campestris*	*Brassica oleracea* var. *gemmifera*	Xanthomonadales	NCPPB 528	Negative
*Clavibacter michiganensis* subsp. *nebraskensis*	*Zea mays*	Micrococcales	DSM 7483	Positive
*Ustilago maydis*	*Zea mays*	Ustilaginales	DSM 14603	n/a

**Table 3:** Pairwise comparisons between dead and lysed cells for each prey and measurement metric. Asterisks indicate statistical significance between predatory behaviour on dead vs. lysed cells within a prey strain, with *p* < 0.05 (*), *p* < 0.01 (**) and *p* < 0.001 (***).

Prey Organism	Displacement	Displacement Ratio	Swarm Rate	Gram Stain
*Pantoea agglomerans*	*		**	Negative
*Pectobacterium atrosepticum*	*	***	**	Negative
*Pseudomonas syringae* pv. *tomato*	*	**	**	Negative
*Rhizobium radiobacter*				Negative
*Xanthomonas campestris* pv. *campestris*				Negative
*Clavibacter michiganensis* subsp. *nebraskensis*				Positive
*Ustilago maydis*	***	**	***	n/a

**Table 4:** Pairwise comparisons of myxobacterial predation metrics for pairs of prey organisms, when provided as dead or lysed prey cells. Asterisks indicate statistical significance between prey strains, with *p* < 0.05 (*), *p* < 0.01 (**) and *p* < 0.001 (***).

		Dead Prey Cells						Lysed Prey Cells					
	Prey Organism	*P. atrosepticum*	*P. syringae*	*R. radiobacter*	*X. campestris*	*C. michiganensis*	*U. maydis*	*P. atrosepticum*	*P. syringae*	*R. radiobacter*	*X. campestris*	*C. michiganensis*	*U. maydis*
Displacement	*P. agglomerans*					***	***		***	*			***
	*P. atrosepticum*		**			***	***		***	**			***
	*P. syringae*				*	***	***			***	**	***	***
	*R. radiobacter*					**	***					**	***
	*X. campestris*					*	***						***
	*C. michiganensis*						***						***
Displacement Ratio	*P. agglomerans*					***	***		***				***
	*P. atrosepticum*		*			**	***		***	***	**		***
	*P. syringae*					***	***			***		***	***
	*R. radiobacter*					*	***					*	***
	*X. campestris*					*	***					*	***
	*C. michiganensis*						***						***
Swarm Rate	*P. agglomerans*		*			***	***		***	**			***
	*P. atrosepticum*		***			***	***		***	***	*		***
	*P. syringae*				**	***	***			***	***	***	***
	*R. radiobacter*					***	***					**	***
	*X. campestris*					*	***					*	***
	*C. michiganensis*						***						***

## Data Availability

The data presented in this study are available on request from the corresponding author.

## Supplementary Materials

The following supporting information can be downloaded at: https://www.mdpi.com/article/10.3390/microorganisms11020398/s1, Table S1: a selection of myxobacteria predation assays from the literature; Figure S1: The effect of nutrients on the outward expansion of myxobacteria spots; Figure S2: behaviour of myxobacteria on dead and lysed prey cells of *Pantoea agglomerans*; Figure S3: behaviour of myxobacteria on dead and lysed prey cells of *Pseudomonas syringae*; Figure S4: behaviour of myxobacteria on dead and lysed prey cells of *Rhizobium radiobacter*; Figure S5: behaviour of myxobacteria on dead and lysed prey cells of *Xanthomonas campestris*; Figure S6: behaviour of myxobacteria on dead and lysed prey cells of *Clavibacter michiganensis*; Figure S7: behaviour of myxobacteria on dead and lysed prey cells of *Ustilago maydis*.
