# Supplementary material for: Myxobacterial Predation: A Standardised Lawn Predation Assay Highlights Strains with Unusually Efficient Predatory Activity
Source: Microorganisms. 2023 Feb 4;11(2):398. doi: 10.3390/microorganisms11020398 (PMC9967850; doi:10.3390/microorganisms11020398)
Supplement: Supplementary file 1 [file microorganisms-11-00398-s001.zip › Supplemental Figures.pptx]

## Slide 1
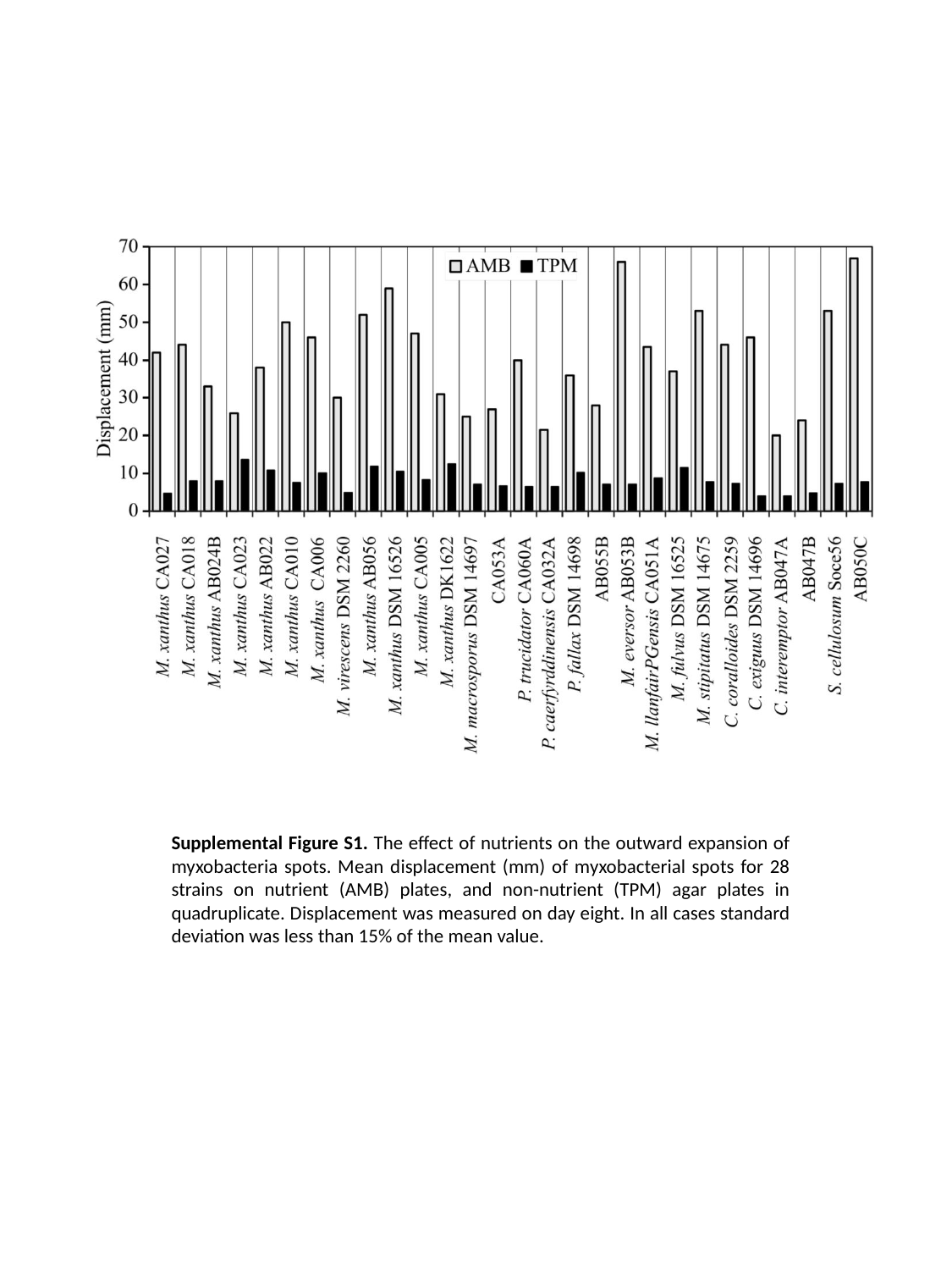

Supplemental Figure S1. The effect of nutrients on the outward expansion of myxobacteria spots. Mean displacement (mm) of myxobacterial spots for 28 strains on nutrient (AMB) plates, and non-nutrient (TPM) agar plates in quadruplicate. Displacement was measured on day eight. In all cases standard deviation was less than 15% of the mean value.

## Slide 2
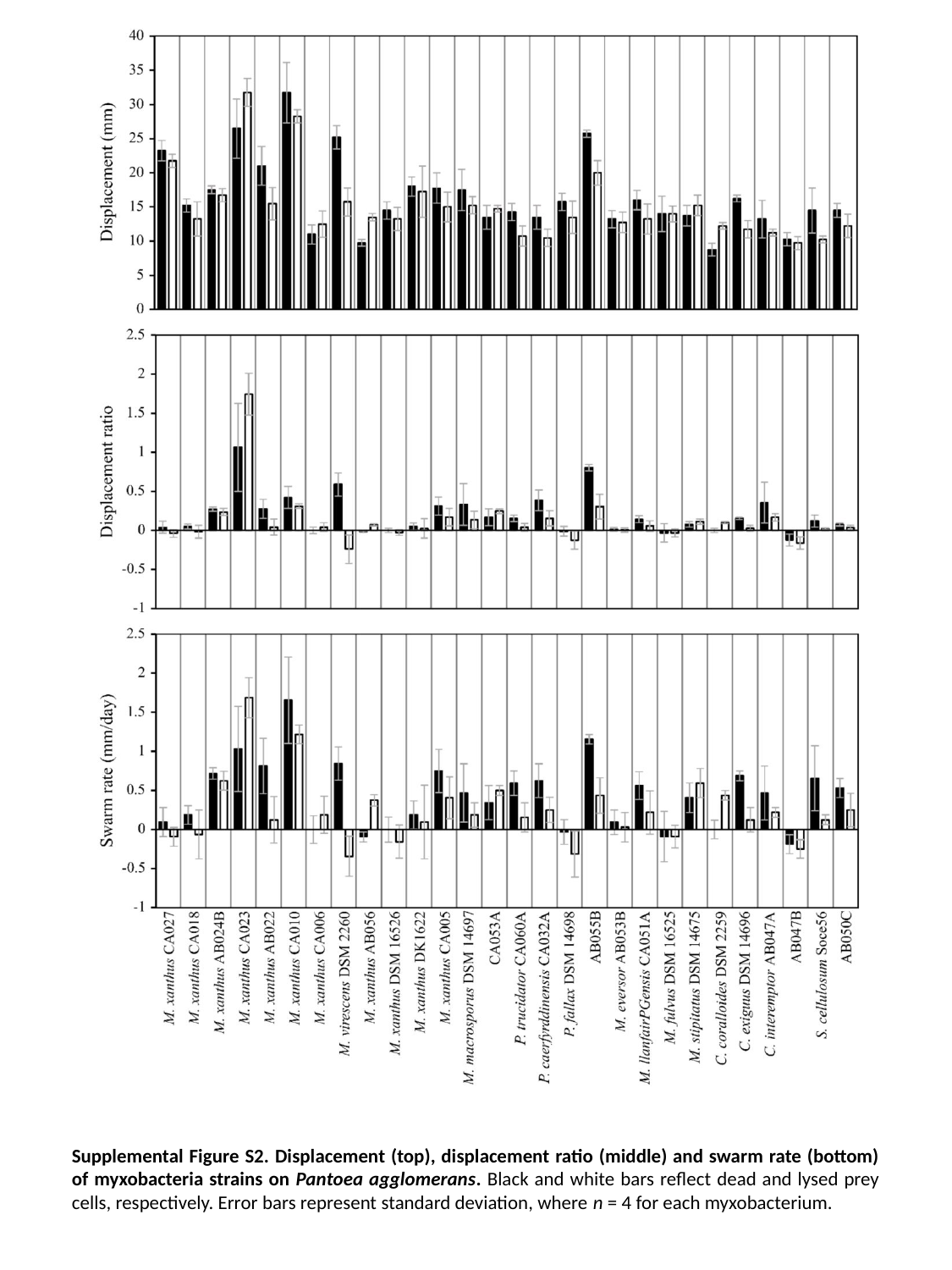

Supplemental Figure S2. Displacement (top), displacement ratio (middle) and swarm rate (bottom) of myxobacteria strains on Pantoea agglomerans. Black and white bars reflect dead and lysed prey cells, respectively. Error bars represent standard deviation, where n = 4 for each myxobacterium.

## Slide 3
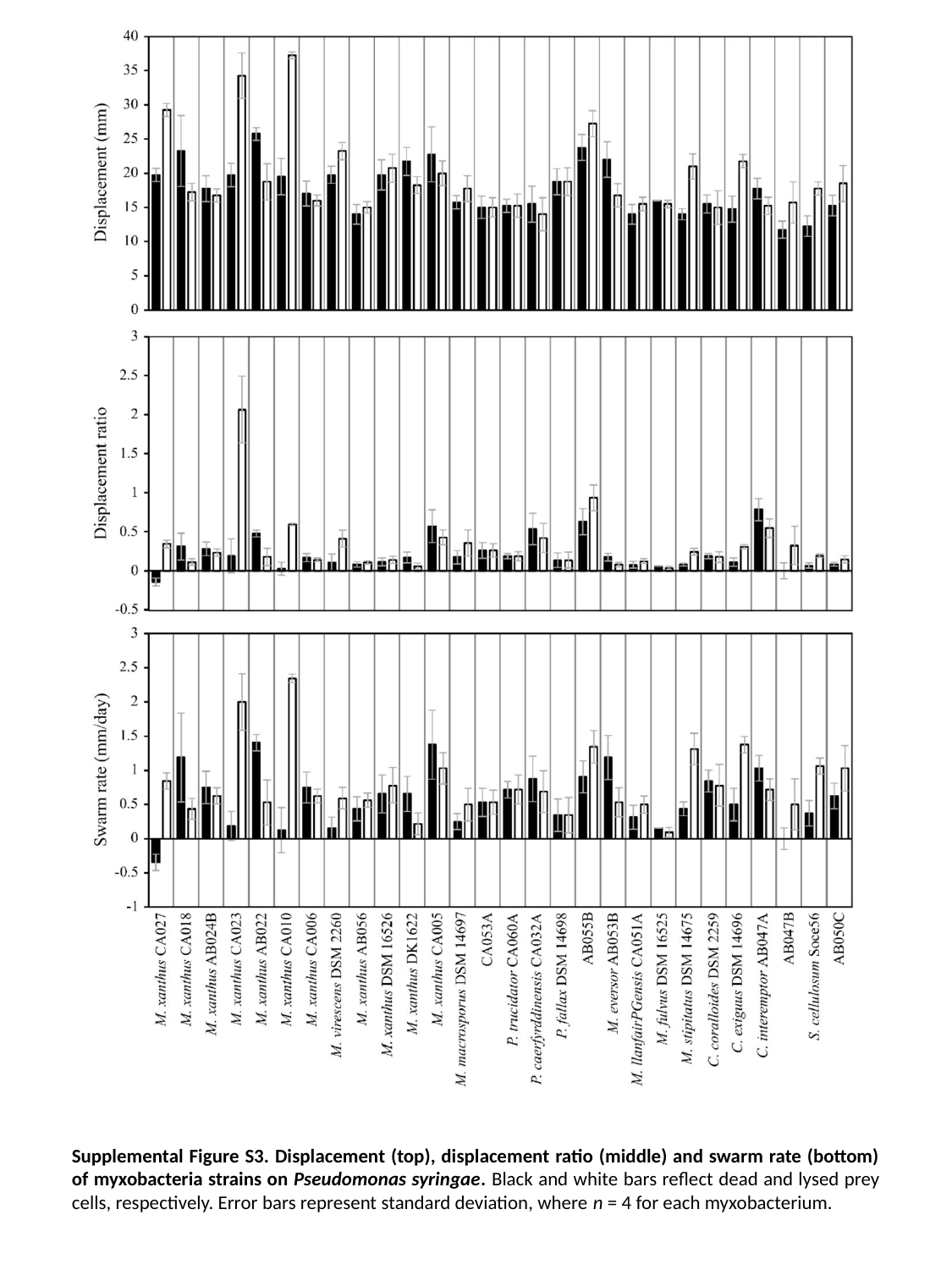

Supplemental Figure S3. Displacement (top), displacement ratio (middle) and swarm rate (bottom) of myxobacteria strains on Pseudomonas syringae. Black and white bars reflect dead and lysed prey cells, respectively. Error bars represent standard deviation, where n = 4 for each myxobacterium.

## Slide 4
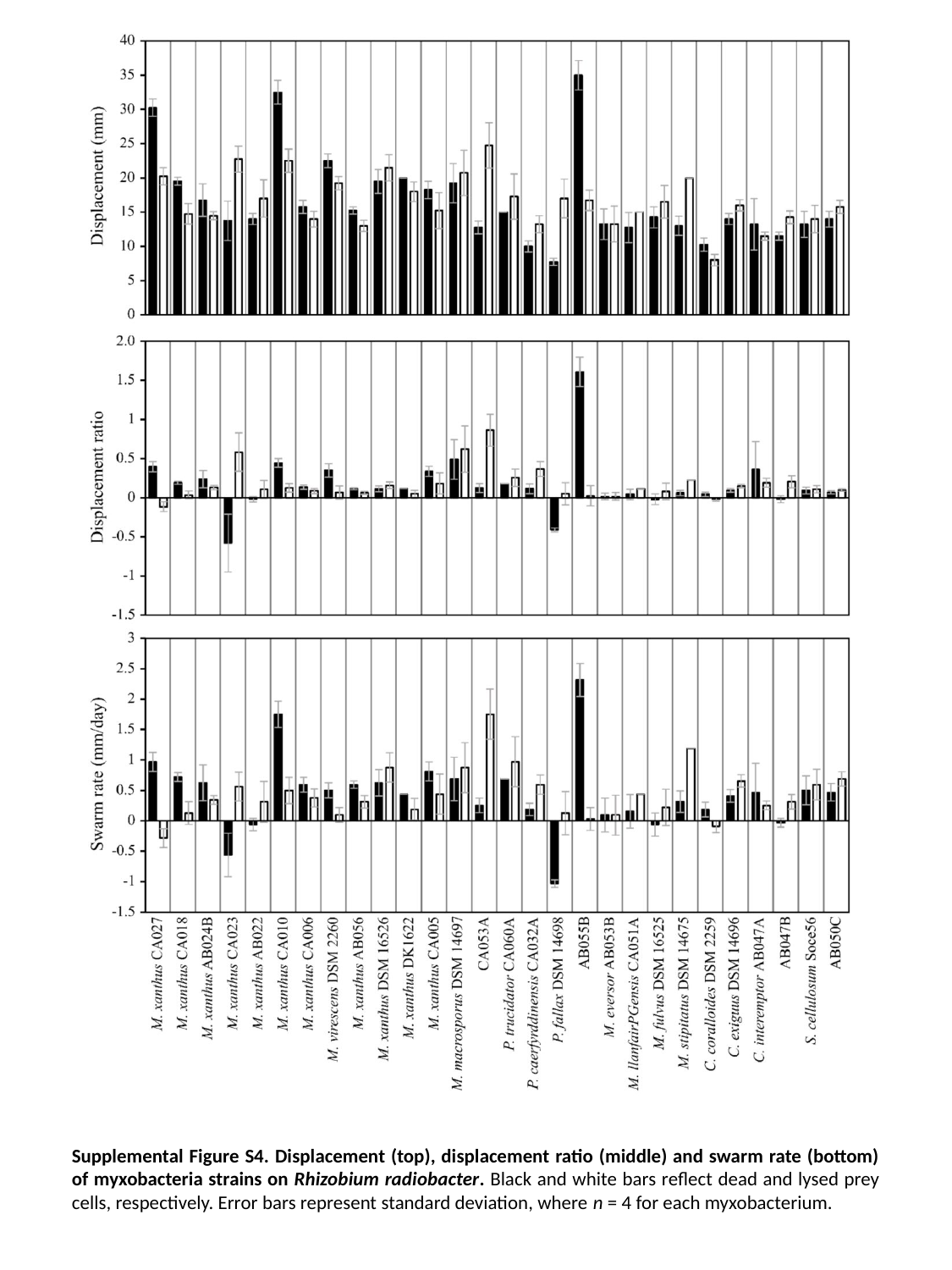

Supplemental Figure S4. Displacement (top), displacement ratio (middle) and swarm rate (bottom) of myxobacteria strains on Rhizobium radiobacter. Black and white bars reflect dead and lysed prey cells, respectively. Error bars represent standard deviation, where n = 4 for each myxobacterium.

## Slide 5
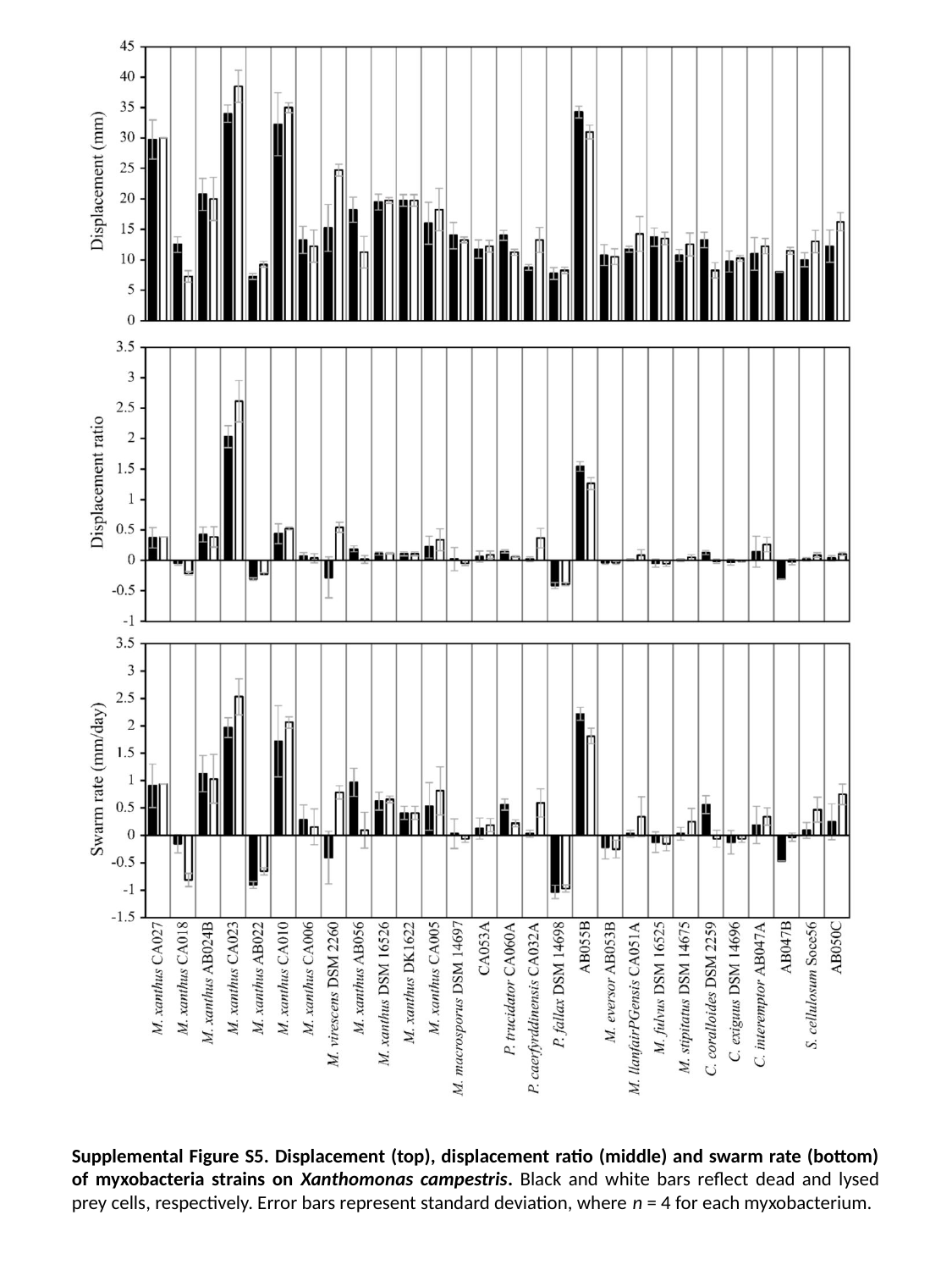

Supplemental Figure S5. Displacement (top), displacement ratio (middle) and swarm rate (bottom) of myxobacteria strains on Xanthomonas campestris. Black and white bars reflect dead and lysed prey cells, respectively. Error bars represent standard deviation, where n = 4 for each myxobacterium.

## Slide 6
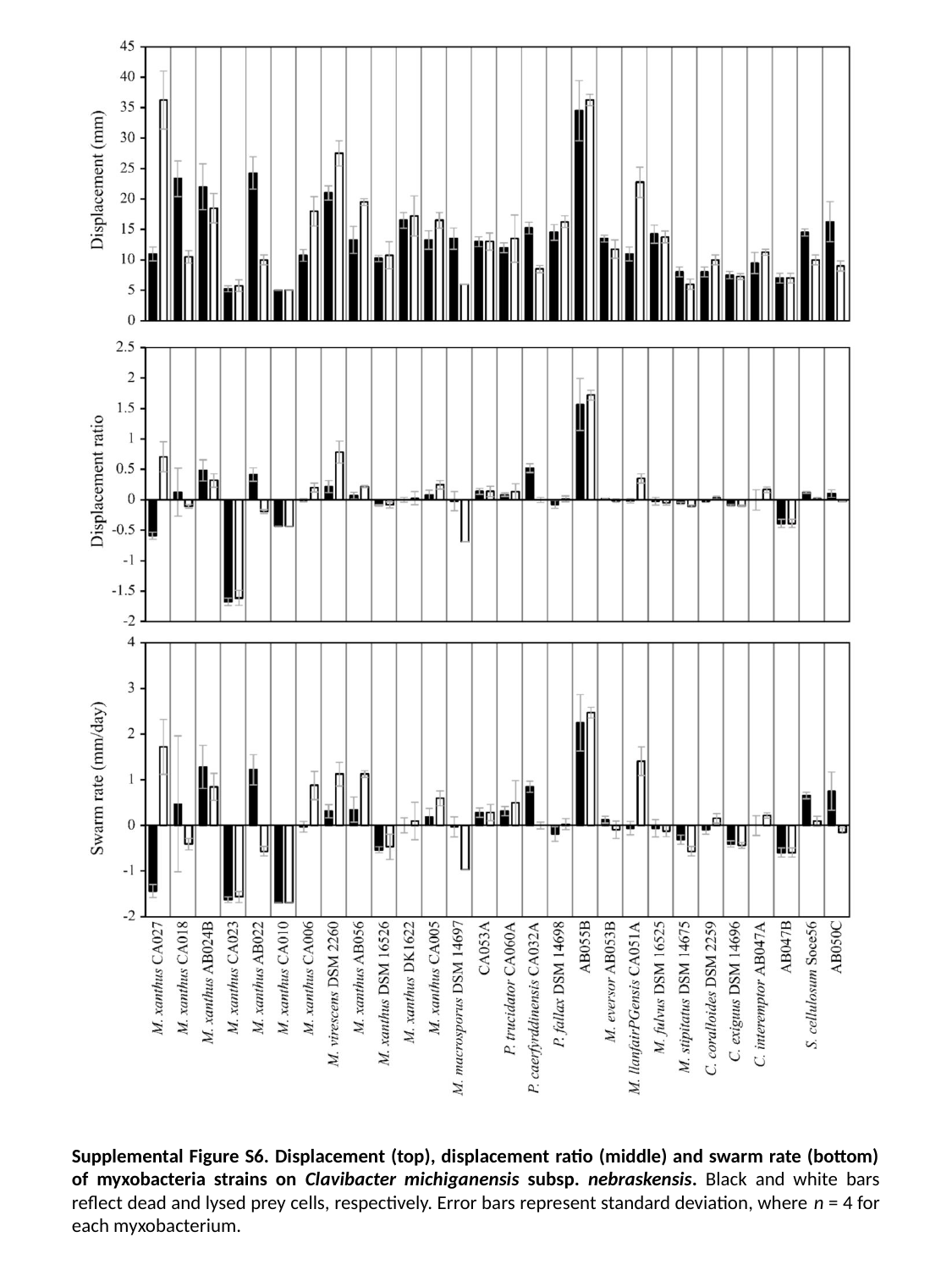

Supplemental Figure S6. Displacement (top), displacement ratio (middle) and swarm rate (bottom) of myxobacteria strains on Clavibacter michiganensis subsp. nebraskensis. Black and white bars reflect dead and lysed prey cells, respectively. Error bars represent standard deviation, where n = 4 for each myxobacterium.

## Slide 7
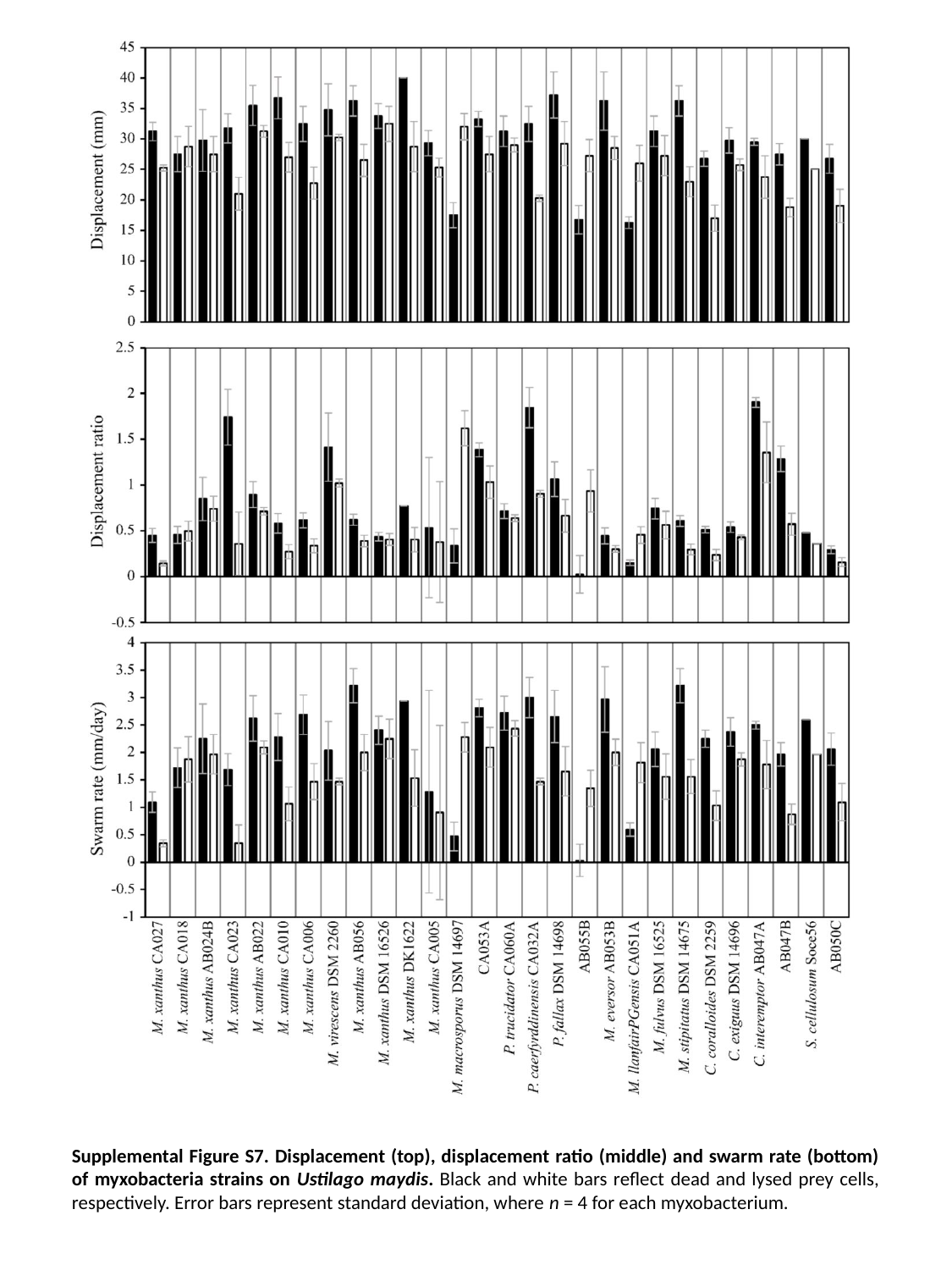

Supplemental Figure S7. Displacement (top), displacement ratio (middle) and swarm rate (bottom) of myxobacteria strains on Ustilago maydis. Black and white bars reflect dead and lysed prey cells, respectively. Error bars represent standard deviation, where n = 4 for each myxobacterium.
